# Integrating Palliative Care into the Indonesia Health System: A Policy Brief to Enhance Accessibility, Quality, and Sustainability

**DOI:** 10.12688/f1000research.171110.1

**Published:** 2025-11-27

**Authors:** Ashar Prima, Dewi Gayatri, Yati Afiyanti, Christantie Effendy

**Affiliations:** 1 Postgraduate Student, Faculty of Nursing, Universitas Indonesia, Depok, West Java, Indonesia; 2 Medical-Surgical Nursing Department, Faculty of health and Pharmacy, Universitas Bani Saleh, Bekasi, West Java, 17113, Indonesia; 3 Basic Science and Fundamendal Nursing Department, Faculty of Nursing, Universitas Indonesia, Depok, West Java, 16424, Indonesia; 4 Maternity and Women Health Department, Faculty of Nursing, Universitas Indonesia, Depok, West Java, 16424, Indonesia; 5 Medical-Surgical Nursing Department, Universitas Gadjah Mada, Yogyakarta, 55281, Indonesia

**Keywords:** Palliative care, health policy, Indonesia, nursing, cancer care, health services accessibility, universal health coverage

## Abstract

**Background:**

Indonesia faces a growing double burden of non-communicable diseases, particularly cancer. The latest data from the Global Cancer Observatory (Globocan) indicates over 408,661 new cases and 242,099 cancer-related deaths in 2022, with a projected 63% increase in the case burden between 2025 and 2040 without strategic intervention. Although a new legal framework through Health Law No. 17 of 2023 and the Minister of Health Decree (KMK) No. HK.01.07/MENKES/2180/2023 has mandated palliative care as an integral component of health services, its implementation still faces significant systemic barriers.

**Policy and Implications:**

This policy brief analyzes the disconnection between the policy mandate and on-the-ground reality, identifying critical gaps in accessibility, healthcare workforce capacity particularly among nursesand financing mechanisms through the National Health Insurance (JKN) program. The failure to effectively integrate palliative care not only causes unnecessary suffering for millions of patients but also burdens the health system with inefficient costs and suboptimal end-of-life care, reflected in the high “financial toxicity” experienced by patients.

**Recommendations:**

We recommend a four-pillar strategy: (1) Formalize and standardize palliative services within the JKN benefits package with a clear financing model to address regulatory ambiguity; (2) Develop a national competency-based palliative education and training strategy for all health workers, with a focus on empowering nurses in primary care; (3) Implement a decentralized and tiered palliative care delivery model centered on Community Health Centers (Puskesmas) to ensure equitable access; and (4) Launch a national public education campaign to destigmatize palliative care and increase awareness.

**Conclusion:**

The integration of palliative care is not merely an option but a strategic and ethical imperative for achieving Universal Health Coverage (UHC) in Indonesia. It is a cost-effective investment to improve patients’ quality of life, support families, and ensure the sustainability of the national health system in facing future non-communicable disease challenges.

## Introduction

Indonesia is facing a pressing public health crisis due to the rising burden of non-communicable diseases (NCDs), especially cancer ( Ministry of Health Republic of Indonesia, 2024). The latest data from the 2022 Global Cancer Observatory (Globocan) presents an alarming picture: there were over 408,661 new cancer cases and 242,099 deaths in Indonesia in a single year. This figure positions cancer as the third leading cause of death in the country, a significant increase from previous data that underscores the urgency of this issue ( Ministry of Health Republic of Indonesia, 2024; World Health Organization, 2025). Furthermore, projections indicate that without robust policy interventions, the burden of cancer cases and deaths in Indonesia will increase by 63% between 2025 and 2040 ( Ministry of Health Republic of Indonesia, 2024; World Health Organization, 2025). This drastic rise not only threatens lives but also the sustainability of the national health system.

This burden is not only clinical but also financial. Cancer ranks second in catastrophic disease financing in Indonesia ( Bray et al., 2024). A study at a national referral hospital revealed a startling fact: 79% of cancer patients experience “financial toxicity” economic hardship due to treatment costs even though they are enrolled in the National Health Insurance (JKN) program ( Bray et al., 2024; International Agency of Research on Cancer, 2025; Ministry of Health Republic of Indonesia, 2024). This directly challenges the assumption that JKN membership has fully resolved health financing issues. It indicates a significant gap in the offered benefits package, especially for comprehensive long-term and supportive care needs.

In facing this challenge, palliative care emerges as an essential and inseparable component. The World Health Organization (WHO) defines palliative care as an approach that improves the quality of life of patients and their families facing the problem associated with life-threatening illness, through the prevention and relief of suffering by means of early identification and impeccable assessment and treatment of pain and other problems—physical, psychosocial, and spiritual ( Brant & Silbermann, 2021; Bray et al., 2024; World Health Organization, 2025). This modern definition shifts the outdated perception that palliative care is only for patients at the end of life. Instead, palliative care is ideally integrated from the time of diagnosis, running concurrently with curative therapies, to ensure patients can live as actively and comfortably as possible at every stage of their illness ( Kang et al., 2024; Kitamura et al., 2023; World Health Organization, 2023).

## Policy outcomes and implications

### The evolving regulatory landscape: From guideline to mandate

The policy framework for palliative care in Indonesia has undergone a significant evolution over the past two decades. Initially, the foundation was laid by the Minister of Health Decree (KMK) No. 812/Menkes/SK/VII/2007, which was a pioneering step in recognizing the importance of palliative care ( Kemenkes, 2007). However, this decree served more as a guideline and lacked full legal binding force, resulting in sporadic implementation dependent on the initiatives of individual health facilities.

A fundamental turning point occurred with the enactment of Law No. 17 of 2023 concerning Health. For the first time in Indonesia’s health legislation history, palliative services were explicitly recognized as one of the pillars of health services that must be provided by the central and regional governments, on par with promotive, preventive, curative, and rehabilitative services ( Kemenkes, 2023). This change elevated the status of palliative care from a mere “option” to a legal “mandate.”

This mandate was further strengthened by KMK No. HK.01.07/MENKES/2180/2023 concerning Guidelines for the Provision of Palliative Services, which replaced the 2007 guidelines ( Kemenkes, 2023). This new guideline is far more comprehensive, detailing the principles of service provision, patient eligibility criteria, and, most importantly, expanding the scope of service delivery to the community level through the concept of “Palliative Homes” as a network of primary health facilities ( Kemenkes, 2022, 2023). This paradigm shift creates a strong legal foundation for the development of structured and equitable palliative services throughout Indonesia, as summarized in Table 1.

**Table 1.** Evolution of palliative care policy in Indonesia (2007-2024).

Criteria	KMK No 812/Menkes/SK/VII?2007	Law No. 17/2023 & KMK No. HK.01.07/MENKES/2180/2023
**Legal Status**	Guideline/Policy	Legislative Mandate
**Nature**	Advisory, not fully binding	Mandatory for Central and Regional Governments
**Definition & Scope**	Tended to focus on terminal-stage patients	Provided from the early diagnosis of life-threatening illness
**Service Provider**	Limited to Type A/B/C/D Hospitals	All healthcare facilities, including Puskesmas and community-based Palliative Homes
**Implication & Gap**	Limited and uneven implementation, dependent on local initiatives	Strong legal mandate, but not yet supported by clear JKN financing mechanisms and adequate human resource capacity

### Implementation gaps: Systemic barriers to access and quality

Despite a robust legal framework, a wide gap exists between the policy mandate and the reality on the ground. This situation can be described as a “mandate without a mechanism” there is a clear legal order, but the structured implementation pathway, especially in terms of financing and human resource development, has not yet been established. This gap manifests in several systemic barriers.

### Geographical and economic disparities

Access to palliative services remains highly unequal. These services are concentrated in a handful of major cities such as Jakarta, Yogyakarta, Surabaya, Denpasar, and Makassar ( Putranto et al., 2017, 2023). This condition creates “palliative care deserts” in most parts of Indonesia, especially in rural and remote areas, where patients and families are left to bear the burden of suffering alone. This disparity is a fundamental obstacle to achieving health equity ( Hidayah, 2024).

### Healthcare workforce capacity crisis

The root cause of the difficulty in developing palliative services is a human resources capacity crisis, especially among nurses, who are the backbone of palliative care. Various studies show inadequate levels of knowledge and attitude; one study reported that 64.8% of nurses had poor knowledge of palliative care ( Wahyuni et al., 2025). This crisis reflects a deeper systemic failure:

a. *Failure of the Education System*: To date, there is no specialist palliative nursing education program in Indonesia ( Wahyuni et al., 2025). Palliative care modules have not been systematically integrated and mandated in the basic medical and nursing curricula ( Agustina et al., 2025). Consequently, graduating health professionals are not equipped with the basic competencies to provide palliative care.
b. *Lack of Professional Recognition*: The crucial role of nurses, from symptom management, psychosocial support, family education, to patient advocacy has not been formally recognized in the form of a clear career path or competency certification ( Malaha et al., 2025). Without recognition and incentives, the motivation to develop expertise in this field remains low.

### The JKN financing puzzle

The ambiguity in JKN financing is the most strategic and crippling upstream barrier. Although the 2023 Health Law mandates palliative services, derivative JKN regulations, including Presidential Regulation No. 59 of 2024, have not explicitly defined the benefits package, eligibility criteria, and tariff schemes for these services ( Susanti & Syofyan, 2025). This “legal ambiguity” creates a negative domino effect: health facilities are hesitant to provide services due to claim uncertainty, and as a result, patients continue to bear significant out-of-pocket costs ( Prasetya et al., 2023). The current policy, which only allows claims with a specific “palliative care” diagnosis, often leads to late referrals when a crisis has already occurred ( Prasetya et al., 2023).

### Socio-cultural barriers and awareness

The final challenge is the low public awareness, and even among some health professionals, of the true concept of palliative care ( Komite Penanggulangan Kanker Nasional, 2019). The persistent stigma that palliative care is synonymous with “giving up” or is only for those nearing death is a major barrier to early referral ( Putranto et al., 2017). Cultural factors, such as the taboo of discussing prognosis and death and a family-centered decision-making model, also require a sensitive and skilled communication approach from the palliative team ( Tampubolon et al., 2021).

### Actionable recommendations

To bridge the gap between policy mandate and implementation, a comprehensive approach touching on four strategic pillars is necessary. These four pillars are designed as a mutually reinforcing ecosystem; the success of one pillar will support the others, and failure in one will weaken the entire system.

***Recommendation 1: Formalize and standardize palliative services within the JKN benefits package***

Addressing the JKN financing ambiguity is the most strategic leverage point. If JKN provides a clear and adequate financing scheme, it will create an economic incentive for health facilities to develop services, which in turn will drive demand for trained health professionals and trigger system-wide change.

***Action***: The Ministry of Health, together with BPJS Kesehatan, must immediately formulate and enact a derivative Minister of Health Regulation (Permenkes) that explicitly defines the scope, criteria, and tariffs for palliative services under the JKN scheme.

Details:

a. ***Scope of Services***: The benefits package must be comprehensive, covering interdisciplinary team consultations, management of pain and complex symptoms, psychosocial and spiritual support for patients and families, and home-based care services.
b. ***Financing Model***: Explore and implement innovative financing models beyond fee-for-service. Options to consider include per diem payments for hospice care or bundled payment models per episode of care for community patients, which can encourage efficiency and holistic care.
c. ***Eligibility Criteria***: Develop and implement evidence-based screening criteria (as indicated in KMK 2180/2023) for the early identification of patients needing palliative services, long before they enter the terminal phase.

***Recommendation 2: Develop a National competency-Based palliative education and training strategy***

A supply of competent health professionals is an absolute prerequisite for quality services. A coordinated national strategy is needed, not just sporadic training sessions.

**Action:** The Ministry of Health, in collaboration with the Ministry of Education, Culture, Research, and Technology and professional organizations (PPNI, IDI), must develop and implement a national strategy for palliative education and training.

Details:

a. ***Curriculum Integration***: Mandate a competency-based palliative care module as part of the core curriculum for undergraduate medical and nursing programs throughout Indonesia.
b. ***National Certification***: Establish a standardized national certification program for medical and nursing personnel providing palliative services. This will create clear competency standards and a recognized career development path. Existing training programs by the Ministry of Health can serve as a basis for this standardization.
c. ***Empowerment of Primary Care Nurses***: Develop a massive and structured special training program for Puskesmas nurses, equipping them with skills to provide basic palliative services, conduct home visits, and act as care coordinators in the community.

***Recommendation 3: Implement a decentralized and tiered palliative care delivery model with a focus on primary care***

To address geographical disparities, the service delivery model must be decentralized, leveraging existing health infrastructure down to the most basic level. Placing Puskesmas and nurses at the center of this model is the most strategic and realistic step, in line with the primary care transformation program being promoted by the Ministry of Health.

**Action**: Regional Governments (Provincial and District/City Health Offices) must adopt and implement a structured, tiered service model to ensure equitable access, consistent with the referral system outlined in national guidelines.

Details: This model can be outlined as in Table 2, which adapts best practices from other developing countries that have successfully implemented community-based palliative care ( Kemenkes, 2023).

**Table 2.** Proposed tiered palliative care delivery model in Indonesia.

Service level	Primary focus	Core team	Key service scope
**Level 1: Puskesmas & Community**	Screening, Basic Care, and Coordination	Palliative-trained Nurse, General Practitioner, Community Health Cadres	Patient identification, basic symptom management (pain, nausea), family education, coordination of home visits, referral to Level 2.
**Level 2: Type C/D Regional Hospital**	Consultation & Complex Case Management	Palliative Consultative Team (Physician, Nurse, Pharmacist)	Receives referrals from Puskesmas, manages more complex symptoms, provides initial psychosocial support, refers stable patients back to Puskesmas.
**Level 3: Type A/B Referral Hospital**	Referral, Education, and Research Center	Full Interdisciplinary Palliative Team (Palliative Specialist, Specialist Nurse, Psychologist, Social Worker, Chaplain)	Manages the most complex cases (refractory pain), interventional procedures, serves as a training and research center, develops clinical guidelines.

***Recommendation 4: Launch a National public education and destigmatization campaign***

Social acceptance and demand for early services can only be realized through massive and sustained public literacy improvement.

***Action:*** The Ministry of Health, through the Directorate of Health Promotion and Germas, must design and launch a comprehensive national campaign to increase understanding and change public perception of palliative care.

Details:

a. ***Key Messaging***: Consistently shift the narrative from “end-of-life care” to “support for the best quality of life at every stage of a serious illness”.
b. ***Communication Channels***: Use a multi-channel approach, including mass media, social media, and the involvement of community figures and religious leaders to deliver culturally relevant and sensitive messages.
c. ***Community Engagement***: Empower civil society organizations, patient support groups, and survivors to share testimonials and real experiences, which are often more impactful than institutional messages ( Eng et al., 2023).

## Discussion

One of the main concerns for policymakers in adopting new health programs is the cost implication. However, strong international and national evidence shows that the early integration of palliative care is a highly cost-effective investment. Various studies have demonstrated that patients who receive early palliative care experience fewer emergency department visits, lower re-hospitalization rates, and fewer aggressive and costly yet futile medical interventions at the end of life. A systematic review even reported potential cost savings of up to USD 20,000 per patient ( Agustina et al., 2025).

Although a study in Indonesia showed that costs and length of stay were initially higher for patients receiving palliative consultation, this finding must be interpreted with caution. This phenomenon is likely due to very late referrals, where the palliative team is called in when the patient is already in a complex crisis condition. The same study also found that when palliative consultation was provided earlier (less than 7 days after admission), the length of stay and costs were significantly lower compared to those who received later consultations ( Putranto et al., 2023). This strengthens the argument that early integration is key to achieving cost efficiency. Thus, financing palliative care through JKN is not an additional burden but a smart strategy to control long-term catastrophic care costs.

Beyond efficiency considerations, providing access to palliative care is an ethical imperative and an integral part of human rights—the right to be free from preventable pain and suffering. The ultimate goal of a health system is not merely to prolong life, but also to ensure that life is lived with quality and dignity. The achievement of Universal Health Coverage (UHC) in Indonesia will never be complete and meaningful if millions of its citizens still have to face serious illness and death in unnecessary suffering. The full integration of palliative care is an essential component of a truly people-centered health system, one that values the dignity and autonomy of patients until the end of their lives ( Susanti & Syofyan, 2025).

## Conclusion

With a now solid legal foundation through the 2023 Health Law and updated technical guidelines, the political momentum for change has been created. Now is the time for concrete action. Translating policy on paper into a reality that can be felt by millions of patients and families across the archipelago requires strong political will and cross-sectoral collaboration. Implementing the four proposed pillars of recommendations formalizing JKN financing, a national education strategy, a tiered service model, and public education will lay the foundation for a robust, equitable, and sustainable palliative care system. Failure to act now will not only betray the legislative mandate but will also exacerbate the health crisis and impose far greater suffering and costs on future generations.

## Data Availability

No data are associated with this article.
