# Supplementary material for: Integrating Palliative Care into the Indonesia Health System: A Policy Brief to Enhance Accessibility, Quality, and Sustainability
Source: F1000Res. 2026 Feb 3;14:1324. Originally published 2025 Nov 27. [Version 2] doi: 10.12688/f1000research.171110.2 (PMC12759270; doi:10.12688/f1000research.171110.2)
Supplement: Peer review history

# Reviewer report: Reviewer response for version 1

**DOI:** 10.5256/f1000research.188659.r437113

**Published:** 2026-01-02

**Authors:** M Agung Akbar

**Affiliations:** 1 Program Studi DIII Keperawatan STIKes Al-Ma’arif, Baturaja, Indonesia

This policy brief is an exceptionally well-crafted and insightful document. It presents a comprehensive, evidence-based analysis of the urgent need to integrate palliative care into Indonesia’s health system. The authors demonstrate strong command of the issue, successfully combining epidemiological data, policy analysis, and practical recommendations. The narrative is coherent, the arguments are persuasive, and the four-pillar strategy provides a realistic, actionable roadmap for policymakers. Overall, the brief makes a significant contribution to national health policy discourse.

 Here are several additional points that could further strengthen the manuscript:

 1.⁠ ⁠The brief would benefit from a concise comparison of Indonesia’s palliative care readiness with countries of similar socioeconomic conditions (e.g., Thailand, Malaysia, Vietnam). This will contextualize Indonesia’s gaps and support the urgency of reform.

 2.⁠ ⁠Adding a short-term (1–2 years), mid-term (3–5 years), and long-term (>5 years) roadmap would help policymakers visualize the sequence of reforms and prioritize actions.

 3.⁠ ⁠Highlighting areas where robust national data are lacking—such as prevalence of unmet palliative needs, would guide future policymakers.

Does the paper provide a comprehensive overview of the policy and the context of its implementation in a way which is accessible to a general reader?

Yes

Is the discussion on the implications clearly and accurately presented and does it cite the current literature?

Yes

Are the recommendations made clear, balanced, and justified on the basis of the presented arguments?

Yes

Reviewer Expertise:

community health and primary health care

I confirm that I have read this submission and believe that I have an appropriate level of expertise to confirm that it is of an acceptable scientific standard.

# Reviewer report: Reviewer response for version 1

**DOI:** 10.5256/f1000research.188659.r437115

**Published:** 2025-12-23

**Authors:** Anisa Purnamasari

**Affiliations:** 1 Universitas Mandala Waluya, Kendari, Indonesia

The paper provides a clear explanation of Indonesia’s palliative care policy history, legal framework, and implementation gaps. Recent local and international literature is cited, demonstrating alignment with global palliative care frameworks (WHO, Worldwide Hospice Palliative Care Alliance). The authors strike a good balance between system-level reforms (e.g., financing and regulation) and operational strategies (e.g., training and community-based care). The manuscript is well written, comprehensive, and policy relevant. It clearly addresses all the criteria. I recommend acceptance.

Does the paper provide a comprehensive overview of the policy and the context of its implementation in a way which is accessible to a general reader?

Yes

Is the discussion on the implications clearly and accurately presented and does it cite the current literature?

Yes

Are the recommendations made clear, balanced, and justified on the basis of the presented arguments?

Yes

Reviewer Expertise:

Nursing

I confirm that I have read this submission and believe that I have an appropriate level of expertise to confirm that it is of an acceptable scientific standard.
